# Motherhood

**Published:** 1909-06

**Authors:** Victoria A. Hardy Duggan

**Affiliations:** San Antonio, Texas


### Contributed.

For Texas Medical Journal.

### Motherhood.

Blest is the mother, who knows the joy
Of the circling arms of her baby boy,
Who spends the day, in "doing things"
To the tune of her own little cherub's wing.

Blest is the mother, who spends the day
In mending garments, torn in play,
Who cuddles the owner of tired feet
And holds him close, while he goes to sleep.

Blest is the mother, who spends the day
In doing things that come her way,
In wiping the tears from the dirty face
And twining each little curl in place.

No woman knows the joy of these things,
The comfort and peace, which each day brings,
Unless her house is full of the noise
Of her own, dear, darling girls and boys!

VICTORIA A. HARDY DUGGAN.

San Antonio, Texas.

